# Discrimination of rosé wines using shotgun metabolomics with a genetic algorithm and MS ion intensity ratios

**DOI:** 10.1038/s41598-020-58193-2

**Published:** 2020-01-24

**Authors:** Mélodie Gil, Christelle Reynes, Guillaume Cazals, Christine Enjalbal, Robert Sabatier, Cédric Saucier

**Affiliations:** 1 Univ Montpellier, SPO, INRAE, Montpellier Supagro, Montpellier, France; 2 Univ Montpellier, IGF, CNRS INSERM, Montpellier, France; 3 Univ Montpellier, IBMM, Montpellier, France

**Keywords:** Metabolomics, Analytical chemistry

## Abstract

A rapid Ultra Performance Liquid Chromatography coupled with Quadrupole/Time Of Flight Mass Spectrometry (UPLC-QTOF-MS) method was designed to quickly acquire high-resolution mass spectra metabolomics fingerprints for rosé wines. An original statistical analysis involving ion ratios, discriminant analysis, and genetic algorithm (GA) was then applied to study the discrimination of rosé wines according to their origins. After noise reduction and ion peak alignments on the mass spectra, about 14 000 different signals were detected. The use of an in-house mass spectrometry database allowed us to assign 72 molecules. Then, a genetic algorithm was applied on two series of samples (learning and validation sets), each composed of 30 commercial wines from three different wine producing regions of France. Excellent results were obtained with only four diagnostic peaks and two ion ratios. This new approach could be applied to other aspects of wine production but also to other metabolomics studies.

## Introduction

Wine is a widely consumed alcoholic beverage with a high commercial value. More specifically, the worldwide consumption of rosé wine has increased by 20% since 2002^1^. Because of its high commercial value, it can become a subject of fraud, and authenticity control is required in order to maintain wine quality and to detect any adulteration^2^. Thousands of molecules can be found in wines, including polyphenols^3^. Recently, more than one hundred polyphenols have been quantified in various rosé wines^4^. They are key components involved in color, taste and quality of wines. Their amount and composition depend on many different factors such as grape variety, geographic origin, winemaking, age. Several methods have already been developed for wine authentication purpose^5^. They can be divided into two categories: metabolite profiling^6–8^ or metabolomic fingerprinting^9,10^. The first one is a targeted analysis focusing on a limited number of representative components while the second one is a non-targeted approach. Both methods were applied to red or white wines. In a previous work^11^, a very fast UPLC-QTOF-MS method was developed to characterize red wines from different grape varieties. One specific ion ratio was used to discriminate commercial red wines from three grape varieties. In this paper, we focused on the influence of the geographic origin of some rosé French wines. The chemical composition of grapes depends on the sum of different environmental conditions, which can be defined as a “terroir” that should influence the grape and wine composition. The goals of this paper were to develop:

- A new and very fast UPLC-QTOF-MS wine metabolomics method with a focus on wine pigments.
- An original statistical method and workflow that allow the robust discrimination of rosés wines according to their origins by using mass spectrometry ion ratio fingerprints.

## Results and Discussions

### UPLC-QTOF-MS analysis

First, a fast UPLC-QTOF-MS method was developed to rapidly acquire high-resolution mass spectra. In accordance with previous work and conclusions, we have used a short gradient instead of isocratic elution conditions or direct injections^11^. It was shown that the last two methods gave limited results probably due to ionization suppression effect. In this work, we chose to work on the positive ionization mode in order to better detect anthocyanins and their derivatives, as they are the main rosé wines pigments. These molecules are present as cationic flavylium ions in acidic pH and are then naturally present as cations in the electrospray source. Minimal sample preparation was used as wines were only centrifuged before analysis.

**Figure 1:**
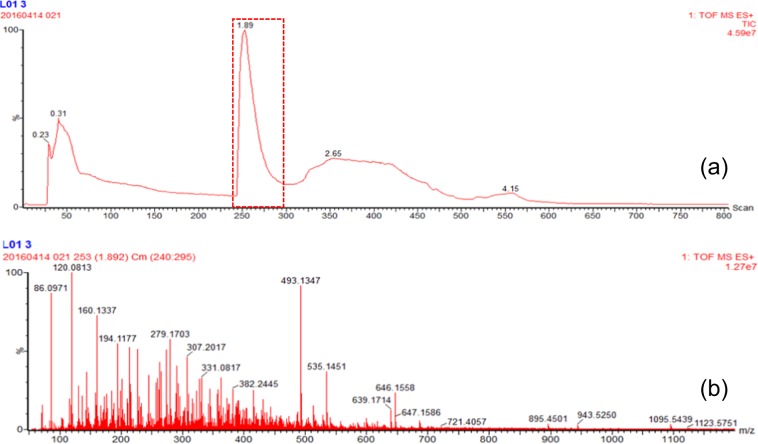
TIC (**a**) and MS spectra (**b**) corresponding to the polyphenols eluting range.

For each wine analysis, the MS spectra was extracted from sum spectra of the Total Ion Current (TIC) between the 240:295 scan ranges. This corresponded to the time range were the polyphenols were eluted (example in Fig. 1).

### Ion ratio discrimination by linear discriminant analysis (LDA) and genetic algorithm

The preprocessing steps described in the Experimental Section led to the identification of 1469 to 3243 (2700 on average) signals among the approximately 40000 ion peaks of the raw mass spectra. The alignment step allowed us to identify 13699 unique ion peaks.

The final objective was to find a very small subset of ion peaks with good discriminant properties within the fingerprints. In order to increase robustness and reproducibility, we made the choice to use ion peak ratios instead of just peak intensities, as ion abundances may change from one injection to another, but their ratios remain stable as shown previously^11^.

The drawback of this strong and original choice is an important challenge with the selection of the best subset of ratios among the 13699 distinct ion peaks obtained after alignment. This lead to approximately 1.9 × 10^8^ possible ratios that could be combined into 2.3 × 10^76^ possible subsets of size 1 to 10 ratios. On an usual desktop workstation, the comprehensive search of the best subset would take 3.4 × 10^66^ years (let us note that the age of the Universe is 14 × 10^9^ years). Hence, a pre-selection of peaks is helpful to ease the fingerprint search.

Furthermore, among the about 14000 identified ion peaks, only a few has been assigned to known components. Yet, a fingerprint based on known components was of better use as it allowed to both infer the wine origin and to understand the differences in terms of components. We chose to focus on polyphenols in our study as these metabolites may be influenced not only by variety but also by abiotic factors. Our research hypothesis is then that these compounds may be used to discriminate the origin of rosé wines. An in-house database of compounds presents in rosé wines –mainly polyphenols- created from previous publications^4,12,13^ was then used to select known ions. Our database comprises 165 components (see Supplementary material) and 72 molecules could be annoted from our list. Hence, a final list of 72 candidates was chosen as a short list for fingerprint identification.

Despite this very important selection, a similar reasoning led to the possibility of 5112 ratios of this 72 ion peaks, which lead to 3.3 × 10^30^ possible subsets of size 1 to 10 and to 4.6 × 10^20^ years of computation for a comprehensive search of the best subset. In this context, usual analysis workflows would fail and powerful heuristic search algorithms are required^14^. We chose a genetic algorithm which has often been used in feature selection contexts^15–17^ including metabolomics biomarkers studies^18,19^. Genetic algorithms are inspired by nature and especially by natural selection and are very useful in such complex optimization issues. Here, the GA was used to find up optimal subsets of peak ratios. The algorithm began with a population constituted of several individuals, which correspond to random potential solutions in the optimization problem. Thus, in our context, the individuals were potential subsets of peak ratios. Then, this population evolved according to three operators: crossover, mutation and selection. Selection was a crucial step allowing to keep the best subsets with regard to their discriminative power (quantified by 2-fold cross-validation use of Linear Discriminant Analysis). Mutation and crossover were run independently from the optimization issue and allowed the solutions to evolve (see Supplementary information).

**Figure 2:**
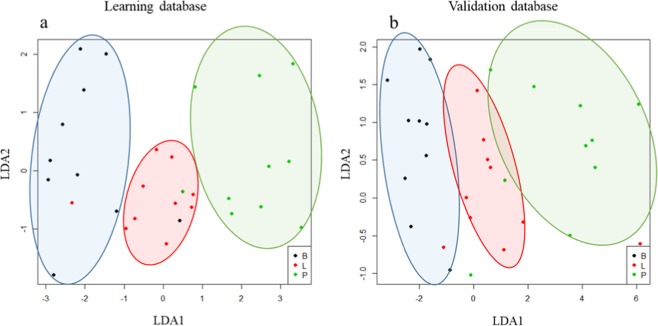
Origin discrimination results on the learning dataset (**a**) and validation dataset (**b**) for rosé wines from Bordeaux (B), Languedoc (L), and Provence (P) gathered using indicative circles.

**Figure 3:**
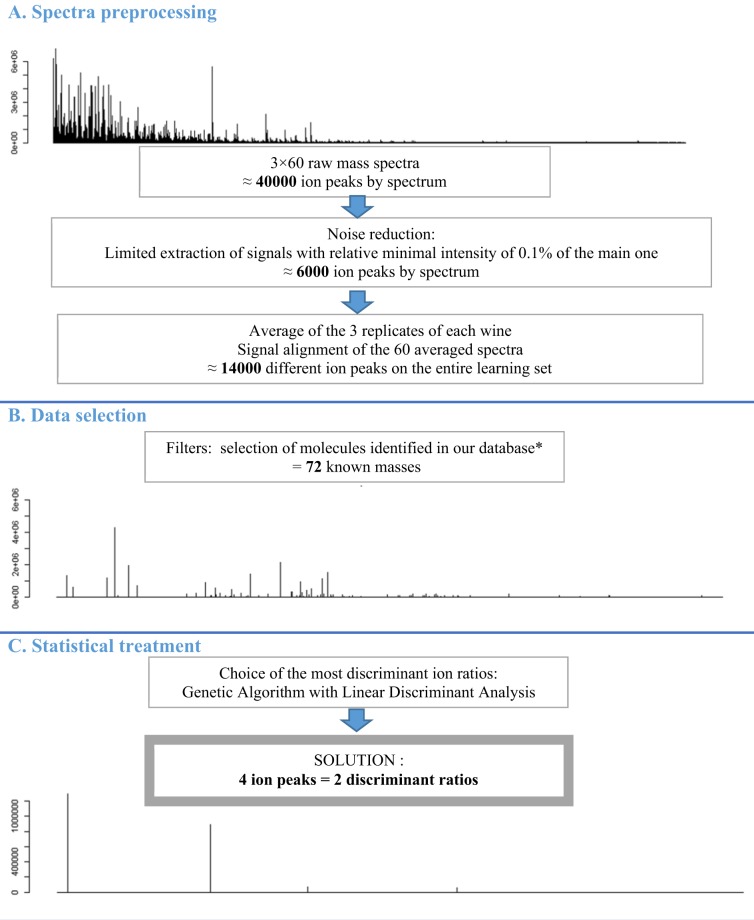
Complete workflow of the discrimination process (*1 –In-house database of molecules in rosé wines created from publications^4,17,18^, details in Supplementary information).

In order to favor solution robustness, the genetic algorithm was run five times and all solutions of the final generations were evaluated through 30 runs of independent linear discriminant analysis with 2-fold cross validation. Solutions were ranked according to their average correct classification rate during the cross-validation process. Then, the solutions with more than 80% of accuracy were tested on an independent validation set (the linear model optimized on the whole learning dataset is applied on the observations in the validation set and accuracy is evaluated). The final selected solution was chosen as the highest correct classification rate on the validation dataset with the lowest number of molecules involved in the fingerprint. This solution contains only four polyphenols, corresponding to two ion ratios. It allows 86.7% accuracy on the learning dataset, 81.7% on average for the cross-validation and 86.7% on the validation dataset. The results are shown in Fig. 2. The entire work flow leading to this solution is summarized in Fig. 3.

In order to assess the adequacy of our approach, we compared it to a very usual method for biomarkers analysis in metabolomics, Random Forests (RF)^14,20–22^. Obviously, there is no embedded method in RF to allow any selection based on ratios. Then, we applied the RF method to the 5112 possible peak ratios. Both number of trees and number of candidates at each split were optimized (see Supplementary information). We built a first RF including all 5112 ratios and used it to identify top ratios (based on variable importance calculations) and ran another RF on the selected ratios. The results obtained with the RF built on the 5112 peaks are provided in Table 1. These are not satisfying results compared to the GA coupled with LDA.

**Table 1:** Summary of classification accuracies both for our approach and Random Forests.

Method	Number of ratios in the model	Correct classification rate on the learning dataset	2-fold cross-validation average correct classification rate	Corect classification rate on the validation dataset
GA + LDA	2	86.7%	81.7%	86.7%
RF	5112	100%	67.3%	70%
RF	6	100%	86.4%	76.7
RF	2	100%	79.4%	50%

Moreover, by studying importance parameters given by the RF algorithm, six ratios were selected (see Supplementary information) and in order to obtain a more comparable model the two top ratios are also used (as we use two ratios in our approach). The results are displayed in Table 1 and show lowest accuracies as long as a trend to overfitting as there is a very big gap between training and validation performances.

### Polyphenols assignment

According to our database, the four phenolic compounds involved in the two discriminant ratios were assigned to vanillic acid, peonidin 3-O-acetyl-Glc-(epi)cat, peonidin 3-O-Glc and (epi)cat-ethyl-(epi)cat isomers. These assignments were determined by comparison with the experimental and theoretical exact masses. The relative error found never exceeded 6.5 ppm (Table 2).

**Table 2:** Experimental and theoretical masses comparison for assignment of discriminant molecules.

Molecule assignment	Experimental m/z	Theoretical m/z	Relative error (ppm)
Vanillic acid	169.0490	169.0501	6.507
Peonidin 3-O-acetyl-Glc-(epi)cat	793.1990	793.1980	−1.261
Peonidin 3-O-Glc	463.1236	463.1240	0.936
(epi)cat-ethyl-(epi)cat (isomers)	607.1810	607.1816	1.060

These molecules have already been identified in rosé wines^4,23,24^. Vanillic acid is a benzoic acid extracted from the solid parts (seeds, skins, stems) of the grape during winemaking that has antioxidant and anti-microbial activities^25^. Peonidin 3-O-Glc and peonidin 3-O-acetyl-Glc-(epi)cat are anthocyanins or anthocyanin derived pigments. It is a family of red grape pigments playing an important role in wine color^26^. Peonidin 3-O-Glc is a monoglucoside, that is one of the most abundant anthocyanin forms in rosé wines after Malvidin 3-O-Glc and its derivatives. On the contrary, peonidin 3-O-acetyl-Glc-(epi)cat is a carbon-carbon adduct with flavanols that forms during wine aging and was detected in very low quantities in rosé wines^4^. (epi)cat-ethyl-(epi)cat is another aging product, formed through oxidation via an acetaldehyde bridging reaction. This results in =CH-CH_3_ (ethyl) bridged flavanols^27^. These polymers gradually accumulate during wine aging due to the gradual chemical oxidation of ethanol in acetaldehyde^28^.

Even if all these polyphenols are present in each group of rosés wines, their relative levels were different and allowed us to discriminate the geographic origin of our wine samples. The use of an independent validation sample set was very important and make our innovative ion ratio approach very promising in our field and for many other applications when discrimination of samples is the objective.

## Conclusion

An original, new and very fast UPLC-QTOF-MS method was developed to analyze more than 6000 ion peaks in a few minutes with minimal sample preparation. An innovative statistical method and workflow was designed and applied to the robust discrimination of rosé wine samples according to their origin. It was compared to Random Forest, a very usual method in biomarker discovery for metabolomics that resulted in lowest accuracy. Indeed, RF benefits from an embedded way of selecting features based on importance measurements. However, this measure is intrinsically univariate (unlike the RF discrimination process which is multivariate) and is not likely to highlight the best synergistic subset of features contrary to our use of GA. This new approach used mass spectrometry and ion ratio fingerprints will be very useful in the future in other fields of metabolomics and sample discrimination.

## Materials and Methods

### Chemicals

All chemicals were of analytical reagent grade. Acetonitrile and formic acid were purchased from Biosolve Chemicals.

Deionized water was obtained from a Direct-Q3 purification system (Millipore).

### Wines and sample preparation

A total of 60 commercial rosé wines were purchased from large retailers. They were selected for their geographic origins (3 different regions of France: Bordeaux, Languedoc, Provence, 20 samples per region), and color range. Wines were from several grape varieties, with unknown wine making processes and from different vintages ranging from 2010 to 2015.

Just after bottle opening, samples of 1.5 mL were prepared and kept in closed plastic Eppendorf at −80 °C. Before analyses, samples were brought to room temperature, centrifuged, and injected in triplicates in a randomized order.

### UPLC-ESI-Tof parameters

Analyses were performed with a Waters Acquity H-Class UPLC system connected to a HD-MS Synapt G2-S mass spectrometer equipped with a Z-Spray source (electrospray ionization ESI). The UPLC system included a vacuum degasser, a quaternary pump (QSM), a cooled autosampler maintained at 10 °C (SM-FTN), and a thermostated column compartment. MassLynx software (version V4.1) was used for instrument control and data processing.

The column used for chromatographic separation was a PLRP-S reversed phase (4000 Å, 50 × 2.1 mm, 5 µm, Agilent Technologies) maintained at 25 °C. The binary mobile phase consisted of Milli-Q water (solvent A) and acetonitrile (solvent B) both acidified with 1% formic acid. The separation was performed at a constant flow rate of 0.6 mL/min, using the following short gradient: 1% B for 1 min; 1–100% B in 0.5 min; 100% B for 0.5 min; 100–1% B in 1.5 min; and reequilibration at 1% B for 2.5 min. The injection volume was 10 µL.

Regarding the detection, the mass spectrometer was operated in the positive ESI mode and data were collected for m/z from 50 to 1800 under the following conditions: capillary voltage, 3.5 kV; cone gas flow, 0 L/h; nitrogen desolvation gas flow, 1000 L/h; desolvation temperature, 350 °C; cone voltage, 60 V.

### Statistic data treatment: from signal preprocessing to discrimination model

All the statistical and preprocessing described in this section has been performed using the R software^29^.

The PROcess R package^30^ has been used to perform spectra preprocessing: baseline substraction and peak extraction. Concerning baseline substraction, the bslnoff function has been used with the loess method and a bandwidth parameter set to 0.1 (all other parameters were set to default values). That is, the function estimates the baseline using the loess (local regression) method with a window of width 0.1, then the function removes this estimated baseline. The peaks extraction was performed through the isPeak function with the following parameters: span = 5, sm.span = 1, zerothrsh = 20000, area.w = 0.05 and SoN = 1.5. It means that each spectrum is first smoothed by using the neareast ‘span’ neighbours. The local variation is estimated using sm.span points. In the window of width ‘span’ the local maximum becomes a potential peak. Then, if the height of this potential peak is ‘SoN’ times higher that the local noise estimated on the other points in the window and if the height of this peak is greater than 1.64 × MAD (smoothed signal in the window), then the peak is considered as validated and output.

Alignment of the obtained peaks was performed using hierarchical clustering with complete linkage^31^. The cut-off threshold has been set in order to minimize the clustering of ions within the same spectrum. After alignment, the average value of peak intensities between technical replicates has been computed and used for further analyses.

Linear Discriminant Analysis^31^ has been used to perform the discrimination of wine origin for a given subset of signals.

The ion peak selection for the final fingerprint was performed with a genetic algorithm^15^. The parameters used for this algorithm are described in the Supplementary information section.

## Supplementary information

Supplementary methods.

Learning dataset.

validation dataset.

## Data Availability

The datasets generated during and/or analysed during the current study are available from the corresponding author on reasonable request.
