# Supplementary material for: Discrimination of rosé wines using shotgun metabolomics with a genetic algorithm and MS ion intensity ratios
Source: Sci Rep. 2020 Jan 24;10:1170. doi: 10.1038/s41598-020-58193-2 (PMC6981237; doi:10.1038/s41598-020-58193-2)
Supplement: Supplementary file 1 — Supplementary methods. [file 41598_2020_58193_MOESM1_ESM.pdf]

Supplementary Methods

Discrimination of rosé wines using shotgun metabolomics with a genetic algorithm and MS ion intensity ratios

Mélodie Gil[1], Christelle Reynes[2], Guillaume Cazals[3], Christine Enjalbal[3], Robert Sabatier[2], Cédric Saucier[1,*]

1- Univ Montpellier, SPO, INRAE, Montpellier Supagro, Montpellier, France

2- Univ Montpellier, IGF, CNRS INSERM, Montpellier, France

3- Univ Montpellier, IBMM, Montpellier, France

[*]Corresponding author at: Université de Montpellier, UMR 1083 INRAE Supagro Sciences pour l'Œnologie, 15 Avenue Charles Flahault, 34000 Montpellier, France. Tel.: +33411759567; fax: +33411759638. E-mail address: cedric.saucier@umontpellier.fr.

Appendix 1. In-house rosé wine compounds database

**Molecule**	**Molecular formula**	**(MH)+ (or M+ for anthocyanins)**
**Organic acids**		
Malic acid	$C_4H_6O_5$	135.0924
Acetic acid	$C_2H_4O_2$	61.029
Lactic acid	$C_3H_6O_3$	91.0395
Tartric acid	$C_4H_6O_6$	151.0243
Gluconic acid	$C_{12}H_{12}O_7$	197.0661
Citric acid	$C_6H_8O_7$	193.0348
**Benzoic acids and derivatives**		
Gallic acid	$C_7H_6O_5$	171.0294
Protocatechuic acid	$C_7H_6O_4$	155.0344
Syringic acid	$C_9H_{10}O_5$	199.0607
Vanillic acid	$C_8H_8O_4$	169.0501
Ethyl gallate	$C_9H_{10}O_5$	199.0606
Ethyl protocatechuate	$C_9H_{10}O_4$	183.0657
Gentisic acid	$C_7H_6O_4$	155.0344
Hydroxybenzoic acid	$C_7H_6O_3$	139.0395
**Hydroxycinnamic acids and derivatives**		
Caffeic acid	$C_9H_8O_4$	181.0501
p-coumaric acid	$C_9H_8O_3$	165.0552
Ferulic acid	$C_{10}H_{10}O_4$	195.0657
Caftaric acid (cis/trans)	$C_{13}H_{12}O_9$	313.056
Coutaric acid (cis/trans)	$C_{13}H_{12}O_8$	297.0611
Fertaric acid	$C_{14}H_{14}O_9$	327.0716
Ethyl caffeate	$C_{11}H_{12}O_4$	209.0814
Ethyl coumarate	$C_{11}H_{12}O_3$	193.0865
3 Caffeoylquinic acid	$C_{16}H_{16}O_9$	355.1029
Sinapic acid	$C_{11}H_{12}O_5$	225.0763
Ferulic acid ethyl ester	$C_{12}H_{14}O_4$	223.0964
GRP	$C_{23}H_{27}N_3O_{15}S$	618.1242

**Stilbenes**		
Resvertrol (cis trans)	$C_{14}H_{12}O_3$	229.0865
Viniferin	$C_{28}H_{22}O_6$	455.1495
Piceid (cis trans)	$C_{20}H_{22}O_8$	391.1393
**Flavonols**		
Quercetin Glc	$C_{21}H_{20}O_{12}$	465.1033
Myricetin Glc	$C_{21}H_{20}O_{13}$	481.0982
Myricetin glucuronide	$C_{21}H_{18}O_{14}$	495.0775
Quercetin glucuronide	$C_{21}H_{18}O_{13}$	479.0826
Quercetin	$C_{15}H_{10}O_7$	303.0505
Kaempferol	$C_{15}H_{10}O_6$	287.0556
Kaempferol Glc	$C_{21}H_{20}O_{11}$	449.1084
Syringetin Glc	$C_{23}H_{24}O_{13}$	509.1295
Isorhamnetin	$C_{16}H_{12}O_7$	317.0661
Syringetin	$C_{17}H_{14}O_8$	347.0766
**Flavan-3-ols**		
Phloroglucinol	$C_6H_6O_3$	127.0395
Catechin	$C_{15}H_{14}O_6$	291.0869
Epigallocatechin	$C_{15}H_{14}O_7$	307.0818
Catechin gallate	$C_{22}H_{18}O_{10}$	443.0978
Galloylated dimer	$C_{37}H_{30}O_{16}$	731.1612
Trimer-1 (1 isomer)	$C_{45}H_{38}O_{18}$	867.2137
Trimers-2 (several isomers)		
(epi)cat-ethyl-(epi)cat-1 (1 isomer)	$C_{32}H_{30}O_{12}$	607.1816
(epi)cat-ethyl-(epi)cat-2 (2 co-eluted isomers)		
Epicatechin	$C_{15}H_{14}O_6$	291.0869
Dimer B2	$C_{30}H_{26}O_{12}$	579.1503
Dimer B1		
Dimer B3		
Dimer B4		
Dimer 1 epigallocatechin B	$C_{30}H_{26}O_{13}$	595.1452
Dimer 1 catechin gallate B	$C_{37}H_{30}O_{16}$	731.1612
Quadrimère cat epi	$C_{60}H_{50}O_{24}$	1155.277

**Dihydroflavonols**		
Astilbin	$C_{21}H_{22}O_{11}$	451.124
Taxifolin	$C_{15}H_{12}O_{7}$	305.0661
**Anthocyanins**		
Pelargonin	$C_{15}H_{11}O_{5}$	271.0607
Delphinidin	$C_{15}H_{11}O_{7}$	303.0505
Cyanidin	$C_{15}H_{11}O_{6}$	287.0556
Petunidin	$C_{16}H_{13}O_{7}$	317.0661
Peonidin	$C_{16}H_{13}O_{6}$	301.0712
Malvidin	$C_{17}H_{15}O_{7}$	331.0818
Malvidin 3,5-diGlc	$C_{29}H_{35}O_{17}$	655.1874
Delphinidin 3,5-diGlc	$C_{27}H_{31}O_{17}$	627.1561
Cyanidin 3,5-diGlc	$C_{27}H_{31}O_{16}$	611.1612
Petunidin 3,5-diGlc	$C_{28}H_{33}O_{17}$	641.1718
Peonidin 3,5-diGlc	$C_{28}H_{33}O_{16}$	625.1769
Pelargonin O Glc	$C_{21}H_{21}O_{10}$	433.1135
Malvidin 3-O-Glc	$C_{23}H_{25}O_{12}$	493.1346
Delphinidin 3-O-Glc	$C_{21}H_{21}O_{12}$	465.1033
Cyanidin 3-O-Glc	$C_{21}H_{21}O_{11}$	449.1084
Petunidin 3-O-Glc	$C_{22}H_{23}O_{12}$	479.1190
Peonidin 3-O-Glc	$C_{22}H_{23}O_{11}$	463.1240
Delphinidin 3-O-acetyl-Glc	$C_{23}H_{23}O_{13}$	507.1139
Cyanidin 3-O-acetyl-Glc	$C_{23}H_{23}O_{12}$	491.1190
Petunidin 3-O-acetyl-Glc	$C_{24}H_{25}O_{13}$	521.1295
Peonidin 3-O-acetyl-Glc	$C_{24}H_{25}O_{12}$	505.1346
Malvidin 3-O-acetyl-Glc	$C_{25}H_{27}O_{13}$	535.1452
Delphinidin 3-O-coumaroyl-Glc (cis- and trans- isomers)	$C_{30}H_{27}O_{14}$	611.1401
Cyanidin 3-O-coumaroyl-Glc (cis- and trans- isomers)	$C_{30}H_{27}O_{13}$	595.1452
Petunidin 3-O-coumaroyl-Glc (cis- and trans- isomers)	$C_{31}H_{29}O_{14}$	625.1557
Peonidin 3-O-coumaroyl-Glc (cis- and trans- isomers)	$C_{31}H_{29}O_{13}$	609.1608
Malvidin 3-O-coumaroyl-Glc (cis- and trans- isomers)	$C_{32}H_{31}O_{14}$	639.1714
Delphinidin 3-O-caffeoyl-Glc	$C_{30}H_{27}O_{15}$	627.1350
Cyanidin 3-O-caffeoyl-Glc	$C_{30}H_{27}O_{14}$	611.1401

Petunidin 3-O-caffeoyl-Glc	$C_{31}H_{29}O_{15}$	641.1507
Peonidin 3-O-caffeoyl-Glc	$C_{31}H_{29}O_{14}$	625.1557
Malvidin 3-O-caffeoyl-Glc	$C_{32}H_{31}O_{15}$	655.1663
(epi)cat-ethyl-peonidin 3-O-Glc (4 isomers)	$C_{39}H_{39}O_{17}$	779.2187
(epi)cat-ethyl-malvidin 3-O-Glc (4 isomers)	$C_{40}H_{41}O_{18}$	809.2293
(epi)cat-ethyl-malvidin 3-O-coumaroyl-Glc (2 co-eluted isomers)	$C_{49}H_{47}O_{20}$	955.2661
Delphinidin 3-O-Glc-(epi)cat	$C_{36}H_{33}O_{18}$	753.1670
Cyanidin 3-O-Glc-(epi)cat	$C_{36}H_{33}O_{17}$	737.1718
Petunidin 3-O-Glc-(epi)cat	$C_{37}H_{35}O_{18}$	767.1823
Peonidin 3-O-Glc-(epi)cat	$C_{37}H_{35}O_{17}$	751.1874
Malvidin 3-O-Glc-(epi)cat (2 isomers)	$C_{38}H_{37}O_{18}$	781.1980
Malvidin 3-O-coumaroyl-Glc-(epi)cat (2 isomers)	$C_{47}H_{43}O_{20}$	927.2348
(epi)cat-delphinidin 3-O-Glc A-F bicyclic	$C_{36}H_{35}O_{18}$	755.1823
(epi)cat-cyanidin 3-O-Glc A-F bicyclic	$C_{36}H_{35}O_{17}$	739.1874
(epi)cat-petunidin 3-O-Glc A-F bicyclic	$C_{37}H_{37}O_{18}$	769.1980
(epi)cat-peonidin 3-O-Glc A-F bicyclic	$C_{37}H_{37}O_{17}$	753.2031
(epi)cat-malvidin 3-O-Glc A-F bicyclic	$C_{38}H_{39}O_{18}$	783.2137
Caftaric-peonidin 3-O-Glc (2 co-eluted isomers)	$C_{35}H_{33}O_{20}$	773.1565
Caftaric-malvidin 3-O-Glc (2 co-eluted isomers)	$C_{36}H_{35}O_{21}$	803.1671
Coutaric-malvidin 3-O-Glc (2 co-eluted isomers)	$C_{36}H_{35}O_{20}$	789.1722
Pyranodelphinidin 3-O-Glc	$C_{23}H_{21}O_{12}$	489.1033
Pyranocyanidin 3-O-Glc	$C_{23}H_{21}O_{11}$	473.1084
Pyranopetunidin 3-O-Glc	$C_{24}H_{23}O_{12}$	503.119
Pyranopeonidin 3-O-Glc	$C_{24}H_{23}O_{11}$	487.124
Pyranomalvidin 3-O-Glc (vitisin B)	$C_{25}H_{25}O_{12}$	517.1346
Carboxypyranodelphinidin 3-O-Glc	$C_{24}H_{21}O_{14}$	533.0931
Carboxypyranocyanidin 3-O-Glc	$C_{24}H_{21}O_{13}$	517.0982
Carboxypyranopetunidin 3-O-Glc	$C_{25}H_{23}O_{14}$	547.1088
Carboxypyranopeonidin 3-O-Glc	$C_{25}H_{23}O_{13}$	531.1139
Carboxypyranomalvidin 3-O-Glc (vitisin A)	$C_{26}H_{25}O_{14}$	561.1244
Pyranodelphinidin 3-O-acetyl-Glc	$C_{25}H_{23}O_{13}$	531.1139
Pyranocyanidin 3-O-acetyl-Glc	$C_{25}H_{23}O_{12}$	515.119
Pyranopetunidin 3-O-acetyl-Glc	$C_{26}H_{25}O_{13}$	545.1295

Pyranopeonidin 3-O-acetyl-Glc	$C_{26}H_{25}O_{12}$	529.1346
Pyranomalvidin 3-O-acetyl-Glc	$C_{27}H_{27}O_{13}$	559.1452
Carboxypyranodelphinidin 3-O-acetyl-Glc	$C_{26}H_{23}O_{15}$	575.1037
Carboxypyranocyanidin 3-O-acetyl-Glc	$C_{26}H_{23}O_{14}$	559.1088
Carboxypyranopetunidin 3-O-acetyl-Glc	$C_{27}H_{25}O_{15}$	589.1194
Carboxypyranopeonidin 3-O-acetyl-Glc	$C_{27}H_{25}O_{14}$	573.1244
Carboxypyranomalvidin 3-O-acetyl-Glc	$C_{28}H_{27}O_{15}$	603.135
Pyranopeonidin 3-O-coumaroyl-Glc	$C_{33}H_{29}O_{13}$	633.1608
Pyranomalvidin 3-O-coumaroyl-Glc	$C_{34}H_{31}O_{14}$	663.1714
Carboxypyranopetunidin 3-O-coumaroyl-Glc	$C_{34}H_{29}O_{16}$	693.1456
Carboxypyranopeonidin 3-O-coumaroyl-Glc	$C_{34}H_{29}O_{15}$	677.1507
Carboxypyranomalvidin 3-O-coumaroyl-Glc	$C_{35}H_{31}O_{16}$	707.1612
p-hydroxyphenylpyranopeonidin 3-O-Glc	$C_{30}H_{27}O_{12}$	579.1503
p-hydroxyphenylpyranomalvidin 3-O-Glc	$C_{31}H_{29}O_{13}$	609.1608
p-hydroxyphenylpyranopeonidin 3-O-acetyl-Glc	$C_{32}H_{29}O_{13}$	621.1608
p-hydroxyphenylpyranomalvidin 3-O-acetyl-Glc	$C_{33}H_{31}O_{14}$	651.1714
p-hydroxyphenylpyranopeonidin 3-O-coumaroyl-Glc	$C_{39}H_{33}O_{14}$	725.187
p-hydroxyphenylpyranomalvidin 3-O-coumaroyl-Glc	$C_{40}H_{35}O_{15}$	755.1976
Catechylpyranopeonidin 3-O-Glc	$C_{30}H_{27}O_{13}$	595.1452
Catechylpyranomalvidin 3-O-Glc (pinotin A)	$C_{31}H_{29}O_{14}$	625.1557
Catechylpyranopetunidin 3-O-acetyl-Glc	$C_{32}H_{29}O_{15}$	653.1507
Catechylpyranopeonidin 3-O-acetyl-Glc	$C_{32}H_{29}O_{14}$	637.1557
Catechylpyranomalvidin 3-O-acetyl-Glc	$C_{33}H_{31}O_{15}$	667.1663
Catechylpyranopetunidin 3-O-coumaroyl-Glc	$C_{39}H_{33}O_{16}$	757.1769
Catechylpyranopeonidin 3-O-coumaroyl-Glc	$C_{39}H_{33}O_{15}$	741.182
Catechylpyranomalvidin 3-O-coumaroyl-Glc	$C_{40}H_{35}O_{16}$	771.1925
Guaiacylpyranomalvidin 3-O-Glc	$C_{32}H_{31}O_{14}$	639.1714
Guaiacylpyranomalvidin 3-O-acetyl-Glc	$C_{34}H_{33}O_{15}$	681.182
Guaiacylpyranomalvidin 3-O-coumaroyl-Glc	$C_{41}H_{37}O_{16}$	785.2082
Syringylpyranomalvidin 3-O-Glc	$C_{33}H_{33}O_{15}$	669.182
Petunidin 3-O-acetyl-Glc-(epi)cat	$C_{39}H_{37}O_{19}$	809.1929
Peonidin 3-O-acetyl-Glc-(epi)cat	$C_{39}H_{37}O_{18}$	793.198
Malvidin 3-O-acetyl-Glc-(epi)cat	$C_{40}H_{39}O_{19}$	823.2086

Pyranopeonidin 3-O-Glc-(epi)cat	$C_{39}H_{35}O_{17}$	775.1874
Pyranomalvidin 3-O-Glc-(epi)cat	$C_{40}H_{37}O_{18}$	805.198
Pyranomalvidin 3-O-coumaroyl-Glc-(epi)cat	$C_{49}H_{43}O_{20}$	951.2348
**Others (aminoacids and alcohols)**		
Tyrosine	$C_9H_{11}NO_3$	182.0817
Tyrosol	$C_8H_{10}O_2$	121.0653
Hydroxytyrosol	$C_8H_{10}O_3$	155.0708
Tryptophol	$C_{10}H_{11}NO$	162.0919
Tryptophan	$C_{11}H_{12}N_2O_2$	203.0821
**Oxidation product**		
GSH	$C_{10}H_{17}N_3O_6S$	308.0916
GSSG	$C_{20}H_{32}N_6O_{12}S_2$	613.1598
2-S-glutathionylcaftaric acid ethyl ester	$C_{25}H_{32}N_3O_{15}S$	646.1554
2-S-glutathionylcaffeic acid	$C_{19}H_{23}O_{10}N_3S$	486.1182

Appendix 2. Genetic algorithms

Genetic Algorithms (GAs) are inspired by nature and especially by natural selection (Goldberg, 1989), they are very useful in complex optimization issues. Here, the GA are used to find up optimal subsets of peak ratios. Thus, it can be considered as a feature selection issue, a field where GAs are widely used (Chandrashekar & Sahin, 2014), especially in metabolomics (Cavill *et al.*, 2009). The algorithm begins with a population constituted of several individuals which correspond to potential solutions in the optimization problem. Thus, in our context, the individuals will be subsets of peak ratios. Then, this population evolves according to three operators described in the next paragraphs: crossover, mutation and selection. Selection is a crucial step allowing to keep the best individuals with regard to the fitness function. Mutation and crossover are run independently from the optimization issue, they allow the exploration of solution space. Then, any GA can be described as follows:

Main steps of a GA:

1. Construction of the first generation

2. Selection

While stopping criteria not met do

3. Crossover

4. Mutation

5. Selection

End

Peak weighting

As the total number of potential solutions is huge (see main manuscript) we decided to associate a weight to each peak in order to favor peaks with two main properties: high values (that is to say high quantities) in at least one group and low within-group variability.

To favor peaks with high values in at least one group, we computed the mean intensities within each group:

$$\bar{x}_j^k = \frac{1}{n_k}\sum_{i\in k} x_{ij}$$

Where x_{ij} is the intensity value of the i-th sample for the j-th ($i \in \{1,2,\ldots,72\}$) peak and k ($k \in \{1,2,3\}$)is the class (here the region). Then we extracted the maximum mean across classes:

$$\bar{x}_j^{max} = \max_{k\in\{1,2,3\}} \bar{x}_j^k$$

Concerning variability, the coefficient of variation of each peak was computed within each group and the average value between groups was kept. In order to introduce a balance between those two criteria, each one was linearly normalized between 0 and 1 across the data. The average value of both normalized criteria was used as the final weight.

Fitness

Genetic algorithms are optimization methods. Hence, a criterion, called fitness, has to be defined in order to quantify the solution quality and to be optimized. As described in the main manuscript, a two-fold cross-validated (2-FCV) accuracy obtained with Linear Discriminant Analysis (LDA) is used to quantify the ability of a subset of peak ratios to discriminate between wine origins (denoted classes in the following). However, in order to avoid overfitting, we favor solutions including few peak ratios. Hence, a penalty term was added to the 2-FCV good classification rate:

$$fitness(S) = accuracy_{CV}(S) - 0.05 \times \frac{card(S) - C_{max}}{1 - C_{max}}$$

Where S denotes a subset of ratios of size $card(S)$ and C_{max} is the maximum number of ratios the user wants to put in a solution. The use of C_{max} only aims at hastening convergence and can be easily changed if the optimal solution reaches the boundary (in our applications C_{max} =10). $accuracy_{CV}(S)$ is the average accuracy obtained by applying LDA to the subset S for 30 runs of 2-fold cross-validation

Initialization

The first population is randomly drawn in order to explore the set of potential solutions while favoring peaks with good inner properties (high intensities and low variability as described earlier). Then, a number of peak ratios is randomly drawn between 1 and C_{max} , and the corresponding number of peaks is chosen following the weights defined earlier. This process is repeated T_{pop} times (in our applications T_{pop} =200).

Selection

This step, based on the fitness values, is defined as in Reeves and Rowe (2003). The individuals are ranked according to their fitness value, the best one having the highest rank. Then, the probability to keep one solution in the next generation is proportional to its rank, so that the sum of probabilities over individuals sums to 1 and that the best individual is twice as likely to be selected as the individual with median rank. Hence, the selection probability of the *k*-th ranked solution is defined by:

$$p_{sel}\left(S_{(k)}\right) = 2 \times \frac{(\ T_{pop} - 2M) \times k + 1}{T_{pop}(3T_{pop} - 4M + 1)}$$

Where M is median of all ranks.

Using those probabilities, T_{pop} -1 solutions are selected. In addition, elitism is applied, that is, the best solution of each generation is automatically introduced in the next one. We obtain a new population of size T_{pop}.

Crossover

The objective of this step is to produce new combinations of the previously retained ratios. It is performed through a usual single-point crossover (Reeves and Rowe, 2003) on ratios and applied to 50% of the individuals of a generation.

Mutation

This step brings the necessary hazard to efficiently explore the solution space. It assures that any point of this space can be reached within a finite number of generations. It is applied to 90% of the individuals of a generation. For each individual to be mutated, three kinds of mutations are possible:

- one of the peak in randomly replaced by another one (still using the weights previously defined) with a probability of 50% ;
- one of the ratios inside the solution is randomly chosen and removed with a probability of 25% ;
- one new ratios is added to the solution as performed in the initialization step with a probability of 25%.

Convergence and stopping criterion

As theoretically proved in Bhandari et al. (1996), two conditions are necessary and sufficient for GA to converge as the number of iterations goes to infinity:

- The best solution in the present population has a fitness value no less than the fitness values of the optimal strings from the previous populations (verified through our selection step).
- Each solution has a positive probability of going to an optimal string within a finite number of iterations (verified through our mutation step).

The designed operators meet those conditions but an infinite number of generations is not praticable so the maximum number of generations is set to 2000. It has been set by studying the evolution of fitness function for several runs and by choosing a number at least twice as large as the number of generations required to reach criterion stability.

Appendix 3. Random Forests

The Random Forest (RF) analysis was performed using the randomForest R package.

Use of importance parameters

In this package two measurements of variable importance are provided (Breiman, 2001): the mean decrease of accuracy (loss of accuracy when a variable is eliminated from trees) and the mean decrease of Gini Index (the same for the Gini Index which is used to choose the best variable and threshold value at each split). They were both used to choose the final set of selected ratios. For example, in the following figure (application to all the ratios), 8 ratios (red points) were selected as having high values of both indicators.

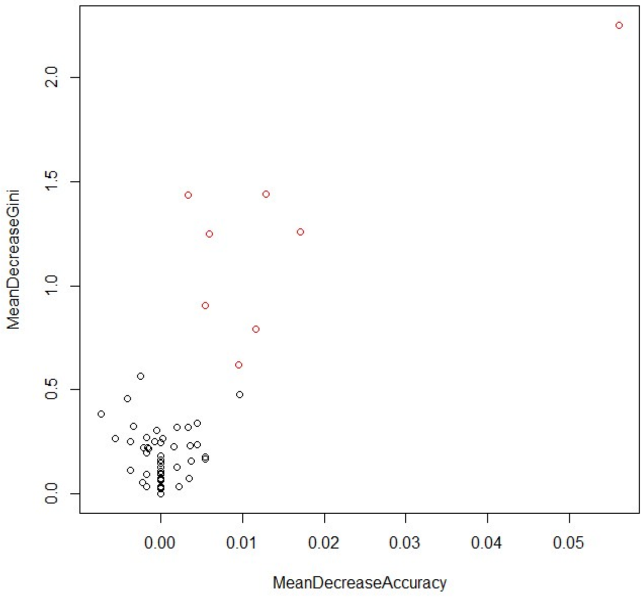

Bibliography

Bhandari, D., Murthy, C.A., Pal, S.K., 1996. Genetic algorithm with elitist model and its convergence. International Journal of Pattern Recognition and Artificial Intelligence 10 (6), 731–747.

Reeves, C.R., Rowe, J.E., 2003. Genetic Algorithms—Principles and Perspectives, A Guide to GA Theory. Kluwer Academic Publishers, London.

Breiman, L. (2001). Random forests. *Machine learning*, *45*(1), 5-32.
